# The complete molecular sequence of chloroplast genome of *Lablab purpureus* (L.) Sweet

**DOI:** 10.1080/23802359.2021.1878958

**Published:** 2021-03-11

**Authors:** Na Li, Ji-Qing Bai, Su Gao, Lei Yang, Jing Li, Shao-Bing Du, Xiao-Ping Wang

**Affiliations:** a College of Pharmacy, Shaanxi University of Chinese Medicine, Xianyang, PR China; b Shaanxi Quality Monitoring and Technology Service Center for Chinese Materia Medica Raw Materials, Xianyang, PR China

**Keywords:** *Lablab purpureus* (L.) Sweet, plastid genome phylogenetic analysis

## Abstract

The complete molecular sequence of chloroplast genome of *Lablab purpureus* (L.) Sweet was firstly assembled and characterized using Illumina sequencing technology. It is 151916 bp in length, with a GC content of 35.4%, and has a typical quadrant structure, including a large single-copy region (LSC), a pair of inverted repeat regions (IRs) and a small single-copy region (SSC), the sequence length is 81132, 53244, 17540 bp, respectively. There are 131 genes in the *L. purpureus* chloroplast genome, including 84 encoding protein genes, 8 *rRNA* genes, and 38 *tRNA* genes. Phylogenetic analysis showed that *L. purpureus* clustered into a large evolutionary clade with three *Vigna* species.

*Lablab purpureus* (L.) Sweet has antioxidant activity, and its extract can promote the proliferation of human gastric epithelial cell line cells (Ren et al. 2019). In this experiment, the complete chloroplast molecular sequence of *L. purpureus* was sequenced and assembled using Illumina second-generation sequencing technology. Generally, the chloroplast genome has a small molecular weight, simple structure, and conservative coverage. At the same time, the rate of gene replacement in the chloroplast genome is low. Except for a few genes located in the inverted repeat region (IR), the others are all single copies, and are less interfered by paralogs genes. They can be used as an important resource for phylogenetic analysis and reflect the kinship between species relationship (Schmid and Gaffron 1966). So after assembly and annotation, sequence analysis was carried out to construct a phylogenetic tree.

The healthy plants and fresh leaves of *L. purpureus* were collected from the Medicinal Botanical Garden of Xianyang City, Shaanxi Province (N 34.316212, E 108.741781), the corresponding specimens were conserved in Shaanxi University of Chinese Medicine (BD-200617005). The genomic DNA was extracted by using a modified CTAB method, and the purity and integrity of the DNA were analyzed by agarose gel electrophoresis. The purity and integrity of the DNA were tested by Nanodrop. After the DNA samples were qualified, they were interrupted by the Covaris ultrasonic disruptor. After end repair, A-tailing, sequencing adapters, purification, PCR amplification, and other steps were applied to completely construct the library. After the library is qualified, the Illumina high-throughput sequencing platform NovaSeq 6000 is used for sequencing. The high-quality reads were obtained, and the clean reads were assembled by the MIRA version 4.0.2 program and MITObim version 1.7 (Brankovics et al. 2016). Annotation of the chloroplast genome with GENEIOUS version 8.0.2 was manually adjusted by comparison with homologous genes of *Vigna unguiculata* (Lonardi et al. 2019) genome (NC_018051.1). Finally, the annotated genome of *L. purpureus* was submitted to GenBank (accession number: MW169030) and the circular genome maps were drawn using OGDRAW (Greiner et al. 2019).

The chloroplast genome of *L. purpureus* sequenced in this experiment is 151916 bp in length, with a GC value of 35.4%, and has a typical quadrant structure. It included a large single copy region (LSC), a pair of inverted repeat regions (IRs) and a small single copy region (SSC), the sequence length is 81132, 53244, 17540 bp, respectively. There are 131 genes in the *L. purpureus* chloroplast genome, including 84 encoding protein genes, eight *rRNA* genes, and 38 *tRNA* genes. The overall GC content of *L. purpureus* chloroplast genome was 35.4%, while the corresponding values of LSC, SSC, and IR regions were 32.9, 41.5, and 28.8%, respectively.

Twenty reference sequences were downloaded from the NCBI website. A total of 21 chloroplast genome sequences with *L. purpureus* were used to construct a phylogenetic tree. The 21 reference sequences were compared by MAFFT software (Katoh and Standley 2013), and then the FastTree software (Liu et al. 2018) was used to construct Phylogenetic tree with the iteration 1000 times. Phylogenetic analysis showed that *L. purpureus* clustered into a large evolutionary clade with three *Vigna* species (Figure 1).

**Figure 1.**
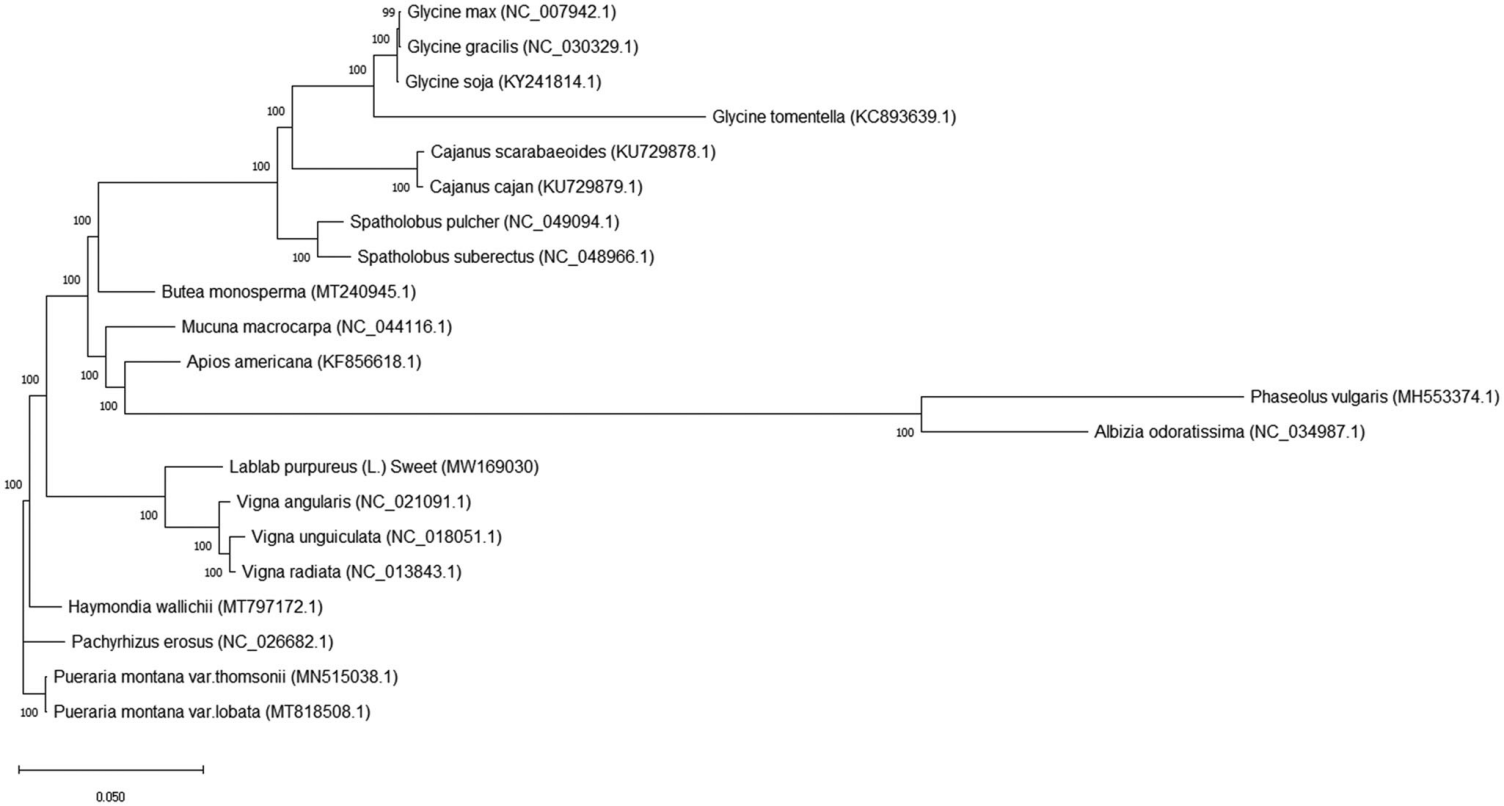
Phylogenetic tree based on 21 complete chloroplast genome sequences. Their accession numbers are after each species names. Numbers in the nodes are the bootstrap values from 1000 replicates.

## Data Availability

The genome sequence data that support the findings of this study are openly available in GenBank of NCBI at (https://www.ncbi.nlm.nih.gov/) under the accession no.MW169030. The associated BioProject, SRA, and Bio-Sample numbers are PRJNA686199, SRP298401, and SAMN17108616, respectively.
